# A case-crossover analysis of the impact of weather on primary cases of Middle East respiratory syndrome

**DOI:** 10.1186/s12879-019-3729-5

**Published:** 2019-02-04

**Authors:** Emma G. Gardner, David Kelton, Zvonimir Poljak, Maria Van Kerkhove, Sophie von Dobschuetz, Amy L. Greer

**Affiliations:** 1 Ontario Veterinary College, University of Guelph, 50 Stone Road E, Guelph, ON N1G 2W1 Canada; 2 World Health Organization, Geneva, Switzerland; 3 Animal Health Service – FAO, Viale delle Terme di Caracalla, Rome, Italy

**Keywords:** Middle East respiratory syndrome, MERS-CoV, Case-crossover, Veterinary public health

## Abstract

**Background:**

Middle East respiratory syndrome coronavirus (MERS-CoV) is endemic in dromedary camels in the Arabian Peninsula, and zoonotic transmission to people is a sporadic event. In the absence of epidemiological data on the reservoir species, patterns of zoonotic transmission have largely been approximated from primary human cases. This study aimed to identify meteorological factors that may increase the risk of primary MERS infections in humans.

**Methods:**

A case-crossover design was used to identify associations between primary MERS cases and preceding weather conditions within the 2-week incubation period in Saudi Arabia using univariable conditional logistic regression. Cases with symptom onset between January 2015 – December 2017 were obtained from a publicly available line list of human MERS cases maintained by the World Health Organization. The complete case dataset (*N* = 1191) was reduced to approximate the cases most likely to represent spillover transmission from camels (*N* = 446). Data from meteorological stations closest to the largest city in each province were used to calculate the daily mean, minimum, and maximum temperature (^ο^C), relative humidity (%), wind speed (m/s), and visibility (m). Weather variables were categorized according to strata; temperature and humidity into tertiles, and visibility and wind speed into halves.

**Results:**

Lowest temperature (Odds Ratio = 1.27; 95% Confidence Interval = 1.04–1.56) and humidity (OR = 1.35; 95% CI = 1.10–1.65) were associated with increased cases 8–10 days later. High visibility was associated with an increased number of cases 7 days later (OR = 1.26; 95% CI = 1.01–1.57), while wind speed also showed statistically significant associations with cases 5–6 days later.

**Conclusions:**

Results suggest that primary MERS human cases in Saudi Arabia are more likely to occur when conditions are relatively cold and dry. This is similar to seasonal patterns that have been described for other respiratory diseases in temperate climates. It was hypothesized that low visibility would be positively associated with primary cases of MERS, however the opposite relationship was seen. This may reflect behavioural changes in different weather conditions. This analysis provides key initial evidence of an environmental component contributing to the development of primary MERS-CoV infections.

## Background

Middle East respiratory syndrome coronavirus (MERS-CoV) is an emerging zoonotic agent that was first isolated in 2012 from a patient hospitalized in Saudi Arabia [1], and has since infected over 2200 people with a 36% case fatality ratio [2]. After an incubation period of 2–14 days [3], the virus causes a disease (Middle East respiratory syndrome, or MERS) characterized by fever, cough, and shortness of breath, which commonly leads to pneumonia and respiratory failure [4]. The virus circulates silently in dromedary camels, the only known reservoir species and zoonotic source of spillover to humans [5]. However, not all primary human cases have documented exposure to dromedaries or their products, such as milk and meat. Although human-to-human community-acquired infections have not been documented, there is evidence that asymptomatic infections of MERS-CoV exist and could be a source of community transmission [6]. Zoonotic spillover from dromedary camels to humans has been documented in the Arabian Peninsula [7]. Subsequent secondary cases can occur after unprotected contact with family members and within healthcare facilities once the primary case seeks medical assistance [8]. While the sizes of MERS-CoV outbreaks have decreased thanks to improved infection control in healthcare settings in affected countries, cases continue to be reported regularly, especially in Saudi Arabia, where surveillance is strong [9]. In order to further reduce cases and prevent human outbreaks, a better understanding of zoonotic transmission of MERS-CoV is needed. A deeper understanding of the epidemiology of primary human cases can inform evidence-based interventions at the level of the community at the animal-human interface.

Zoonotic modes of MERS-CoV transmission have not yet been definitively determined. MERS-CoV in dromedary camels causes a mild upper respiratory infection with no documented viremia [10], and therefore droplet or aerosol transmission by close camel contact is most likely. However, transmission through contaminated milk, meat, and urine is possible, although the contribution of camel products cannot currently be estimated due to a lack of scientific evidence.

The effects of weather and environmental conditions on respiratory diseases with similar modes of transmission (direct contact or droplet), such as influenza and respiratory syncytial virus, have been documented. Temperature and humidity are associated with transmissibility of influenza virus [11], and the seasonality of both influenza and respiratory syncytial virus is linked to these two factors [12]. Air quality is also associated with respiratory infections. Air pollution has been linked to pneumonia and acute lower respiratory infections [13, 14], while dust storms are associated with infectious respiratory disease by acting both as a carrier of pathogens and increasing airway susceptibility to infection [15]. The risk of acquiring primary MERS may be influenced by changes in weather conditions in two ways. First, weather conditions may affect the viability and persistence of the virus in the environment and therefore its transmissibility [11, 16]. Secondly, weather influences behaviour, and it is plausible that the likelihood of people contacting camels depends on environmental conditions. Seasonal or meteorological patterns of primary MERS-CoV infections have yet to be explored.

This study examined whether meteorological conditions were associated with the development of known primary MERS-CoV infections using a case-crossover study design. Case-crossover studies are designed so that exposures during a period of interest before a case are compared to exposures during control periods before or after the case. In this regard, case-crossover studies answer the question “why now?” as opposed to “why these subjects?” [17]. The design is well suited for rare diseases with short incubation periods such as MERS-CoV. The effect period, that is, the period of time after the proposed “trigger”, typically has a degree of uncertainty [17], leading to exposure windows with intervals of biological relevance to the outcome of interest. For infectious diseases, this would equate to the incubation period [18]. Furthermore, with appropriate selection of referent windows, the case-crossover design controls for confounding effects of temporal fluctuations such as climatic and livestock-associated seasons (e.g. the dromedary breeding cycle) [19]. By comparing weather conditions immediately before MERS cases to weather conditions at other times, this study aimed to identify environmental factors that are associated with primary human MERS in Saudi Arabia.

## Methods

### Case data

The World Health Organization (WHO) maintains a list of all human laboratory confirmed cases of MERS-CoV. Publicly available case data from January 2015–December 2017 were obtained. Case data prior to 2015 were excluded due to a lack of standardized data collection prior to 2015 [20].

**Fig. 1:**
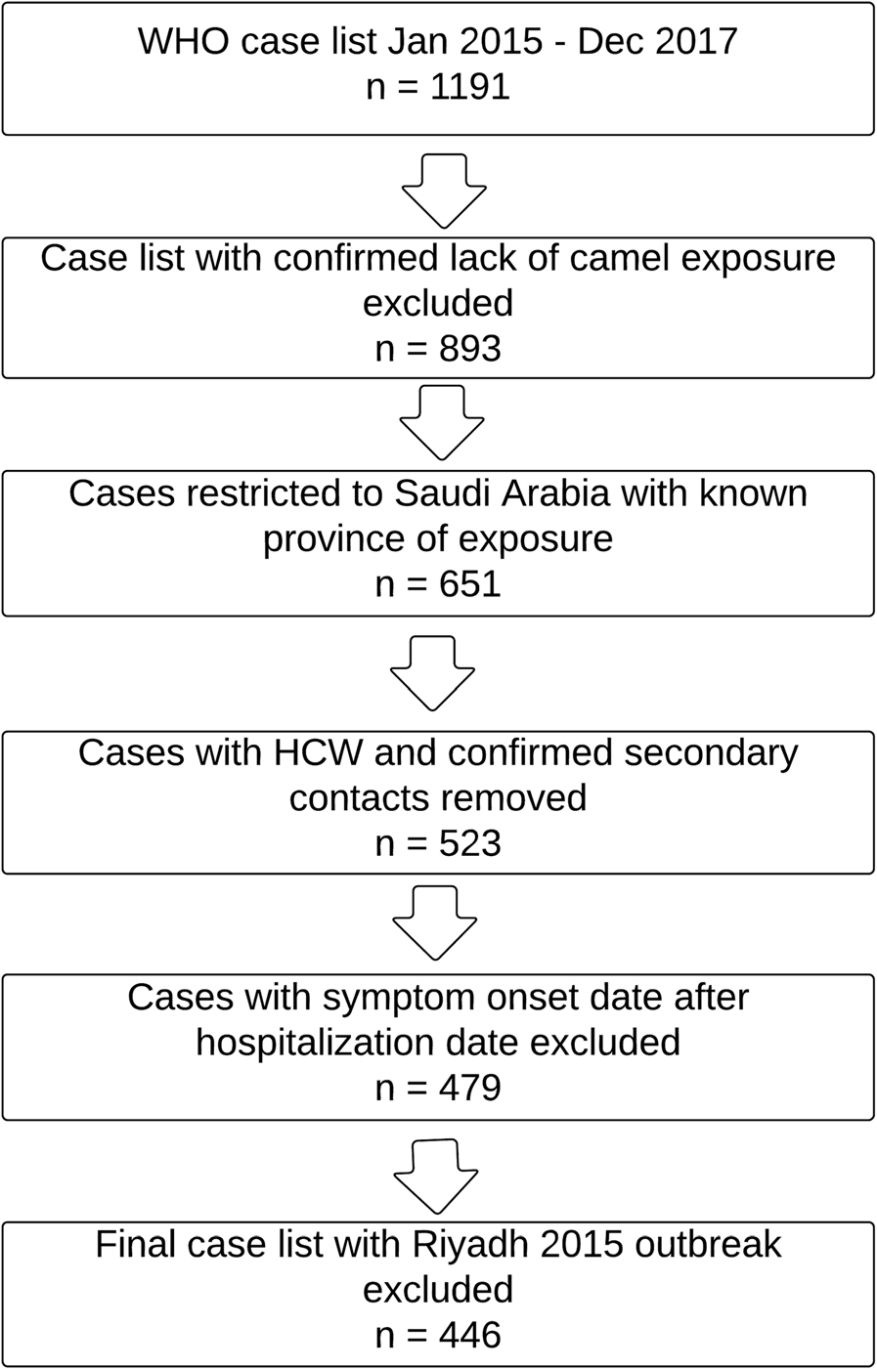
Flowchart of case inclusion/exclusion process to arrive at a subset of primary MERS cases. *HCW = healthcare worker

A MERS case was defined throughout the study period as “A person with laboratory confirmation of MERS-CoV infection irrespective of clinical signs and symptoms” [21]. Of the 1191 confirmed cases with onset dates between January 2015 – December 2017, 298 cases were removed where exposure to camels and camel products were known not to have occurred. Geographically, cases were restricted to those reported from Saudi Arabia, where the province of exposure was provided (*n* = 651). Cases that were likely primary cases were retained by excluding healthcare workers and cases with documented contact with known MERS cases (*n* = 128). Cases were further removed where symptom onset date was after hospitalization date (*n* = 44). Of the remaining cases (*n* = 479), 10 (2.1%) had missing symptom onset dates. To retain these ten cases, the median time between symptom onset date and lab confirmation date was calculated (6 days) and subtracted from the lab confirmation date to obtain an estimate of the symptom onset date. Visual inspection of the timeline of retained cases identified a spike from Riyadh province around August 2015, which corresponds to a documented MERS-CoV outbreak in the city of Riyadh from July–September 2015. Data from a published report of the outbreak contained weekly counts of primary and secondary cases [22]. These weekly counts were compared with the case list for this analysis and thirty-two secondary cases associated with the Riyadh outbreak were removed. The final number of retained cases fitting the primary case definition was 446 (Fig. 1). For the purposes of the descriptive results, age groups were chosen for ease of reading while still providing a visualization of the distribution, and according to age categories provided by the Statistical Yearbook of the General Authority for Statistics of the Kingdom of Saudi Arabia, which was used for standardization.

### Meteorological data

**Fig. 2:**
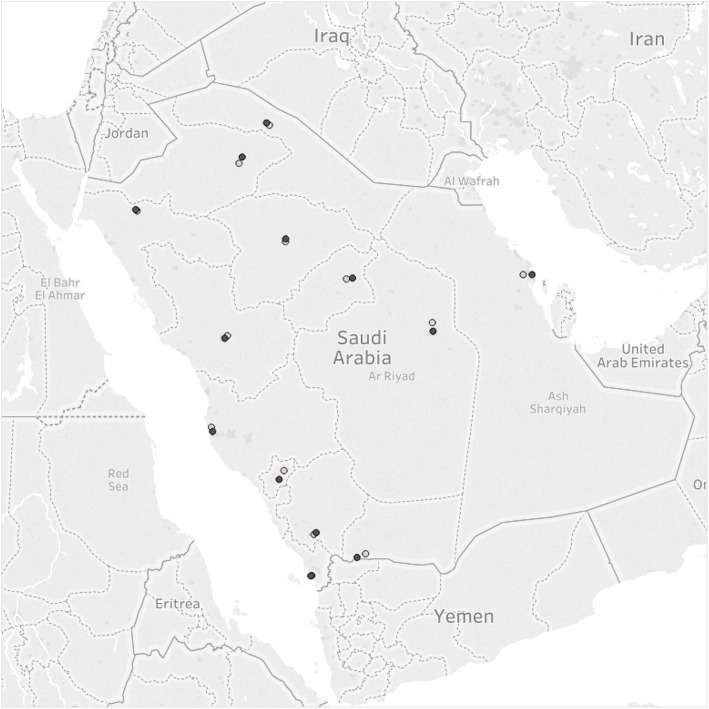
Map of Saudi Arabia with the largest city in each province by population (closed circle), and nearest weather station (open circle). The map was created using Tableau© Desktop 10.5 using built-in base maps

Meteorological stations closest to the largest city in each province were identified by a numeric identifier and location using Google Earth [23] (Fig. 2). Meteorological data were obtained from the NOAA global hourly index [24]. The daily mean, minimum, and maximum temperature, wind speed, and visibility were calculated. Relative humidity was calculated using temperature and dew point data [25].

### Case-crossover analysis

A case-crossover design was used to explore the associations between primary MERS cases and preceding meteorological conditions [17, 26]. Each case’s exposure status on individual days before disease onset (the exposure window) was compared to the exposure status on different days during a control period. Under the assumption that weather effects on virus transmission were immediate, the exposure window, that is, the time lag between weather events and disease onset, was set to be equal to the MERS incubation period of 2–14 days [3]. Univariable conditional logistic regression was used to assess statistical associations between cases and weather variables on each day within the case and control exposure windows. Associations with *p* < 0.05 were considered statistically significant. A time-stratified design was used, with a 28-day strata length with random bi-directional controls matched by day of the week. Using a 28-day time window provides at least three control days for each case exposure day while minimizing bias introduced from seasonal changes [27]. Temperature and humidity variables were categorized into tertiles calculated within each time stratum. Wind speed and horizontal ground visibility were categorized into two groups within each stratum with the median as cutoff. Therefore, there is no single threshold for each weather variable, but rather “low”, “medium” and “high” are determined according to the measurements in each stratum. Statistical analyses were conducted using STATA 15.0 (STATA Corporation, College Station, TX).

## Results

### Descriptive results

**Fig. 3:**
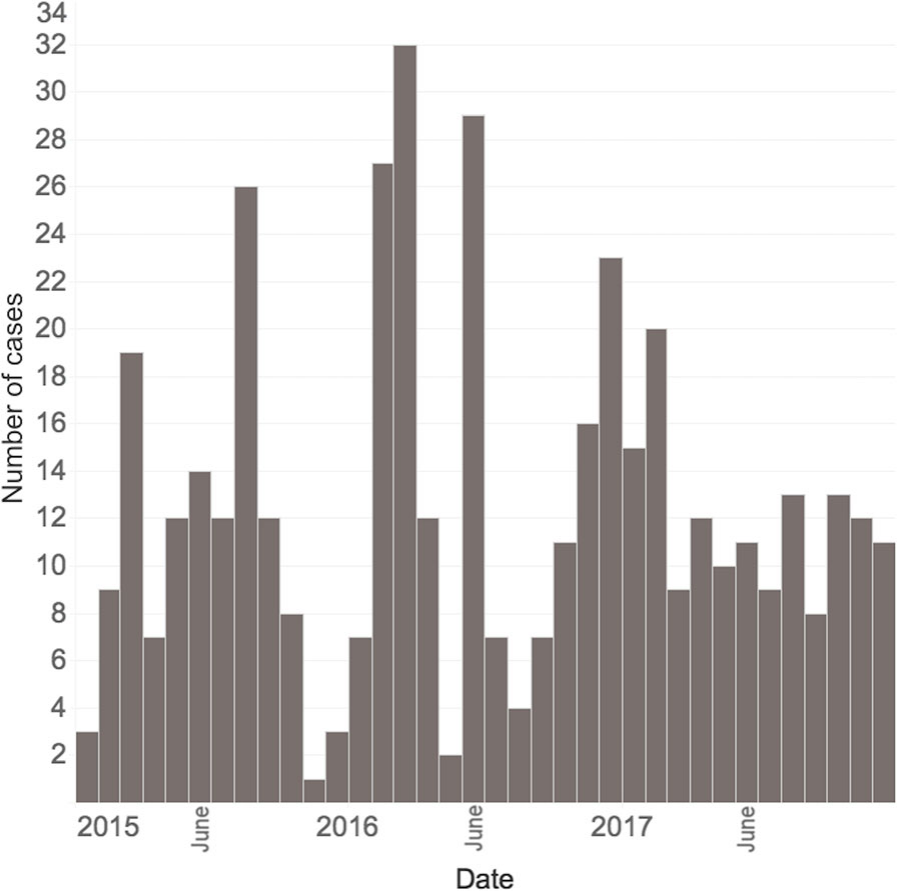
The distribution of 446 primary MERS-CoV cases in Saudi Arabia, 2015–2017 by month and year of symptom onset

Four hundred and forty-six cases of MERS-CoV in Saudi Arabia with symptom onset dates between January 2015 – December 2017 were included in the analysis. Table 1 presents the case counts as well as crude and age- and sex- standardized rates by province, sex, and age group. All 13 provinces in Saudi Arabia reported cases during this 3-year period. Riyadh province had the highest count of reported cases with 185 cases (42%), although Qasseem had the highest cumulative incidence (3.8 cases per 100,000 people), followed by Riyadh (2.54 cases per 100,000 people). The median age of cases was 58 years (range, 15–98), and 79% of cases were male. Age and sex proportions are similar to figures reported for primary cases in previously published literature [28]. Figure 3 presents the case count by month from 2015 to 2017 for the entire country. Cases were reported in every month of the year, although no clear seasonality is apparent.

**Table 1:** Cumulative case counts and rates of MERS in Saudi Arabia from 2015 to 2017

Population	Cases (%)	Cases per 100 k Crude	Cases per 100 k adjusted^a^
**Province**			
Asir	20 (4.4)	0.93	0.90
Baha	4 (0.9)	0.86	0.69
Eastern	53 (11.9)	1.16	1.24
Hail	17 (3.8)	2.50	2.39
Jawf	3 (0.7)	0.82	0.89
Jizan	2 (0.4)	0.13	0.14
Madinah	23 (5.1)	1.13	1.02
Makkah	54 (12.1)	0.68	0.66
Najran	24 (5.4)	4.42	4.66
Northern	2 (0.4)	0.56	0.58
Qasseem	52 (11.7)	3.80	3.80
Riyadh	185 (41.5)	2.43	2.54
Tabuk	7 (1.6)	0.90	1.00
TOTAL	446 (100)	1.46	1.46
**Age**			
< 20	2 (0.4)	0.02	0.02
> 20 < 65	289 (64.8)	1.47	1.43
> 65	155 (34.8)	17.27	17.27
**Sex**			
Male	352 (78.9)	2.05	1.99
Female	94 (21.1)	0.70	0.72

^a^Age and sex adjusted rates were calculated using data from the 2015 Statistical Yearbook of the General Authority for Statistics of the Kingdom of Saudi Arabia

### Analytical results

**Fig. 4:**
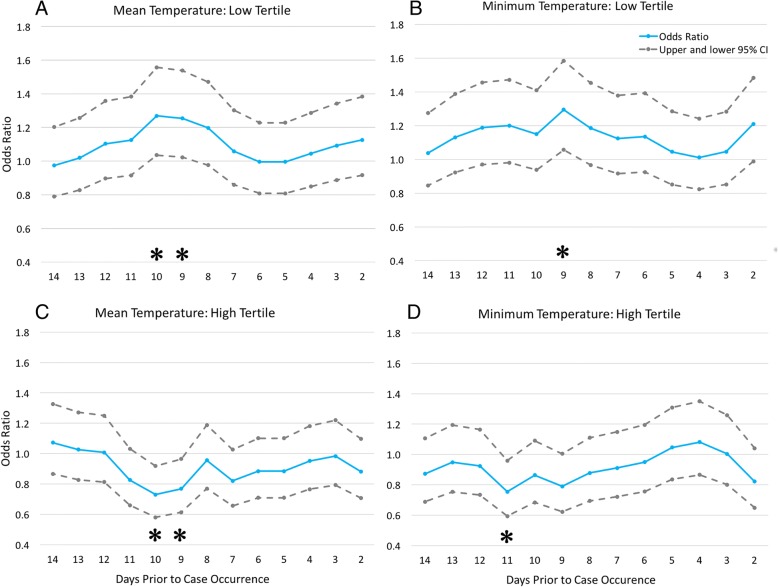
Daily mean and minimum temperature and risk of primary MERS by province in Saudi Arabia. Odds ratio (solid line) and 95% confidence limits (dashed lines) are plotted on the Y-axis, while time lags preceding case occurrence are plotted on the x-axis. The odds of primary MERS is increased with low temperature at 9 and 10 day lags (**a** &**b**), while the odds of primary MERS are decreased with high temperatures at 10 and 11 day lags (**c** & **d**). Asterisks indicate statistically significant odds ratios on corresponding days

Temperature and humidity conditions were associated with case occurrence 8–11 days later. The odds of a MERS case 9 days after low minimum temperatures was 1.29 (95% Confidence Interval [CI], 1.06–1.58) higher than after control days, while low mean daily temperature was similarly associated with cases at 9 (OR, 1.25; 95% CI, 1.02–1.54) and 10 day lags (OR, 1.27; 95% CI, 1.04–1.56) (Fig. 4). Conversely, high minimum, maximum, and mean temperatures were protective at similar lag days. For example, the odds ratios of MERS cases for the high mean daily temperature was 0.77 (95% CI, 0.61–0.96) with a 9-day lag, and 0.73 (95% CI, 0.58–0.92) with a 10-day lag.

**Fig. 5:**
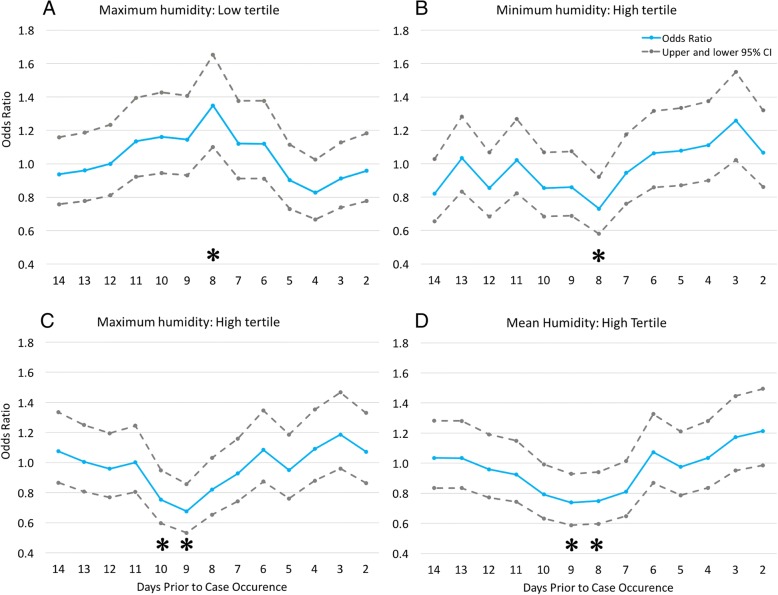
Daily humidity variables and risk of primary MERS by province in Saudi Arabia. Odds ratio (solid line) and 95% confidence limits (dashed lines) are plotted on the Y-axis, while time lags preceding case occurrence are plotted on the x-axis. The odds of primary MERS is increased 8 days after relatively low humidity (**a**), while the odds of primary MERS are decreased with higher humidity at 8–10 day lags (**b-d**). Asterisks indicate statistically significant odds ratios on corresponding days

Humidity followed a similar pattern to temperature. When maximum daily humidity was low 8 days earlier, the odds ratio for a MERS cases was 1.35 (95% CI, 1.10–1.65). High humidity was associated with fewer cases across all three daily measurements (Fig. 5). For example, the odds ratios of cases for high maximum daily humidity was 0.68 (95% CI, 0.53–0.86) and 0.75 (95% CI, 0.60–0.95) at 9- and 10-day lags, respectively.

**Fig. 6:**
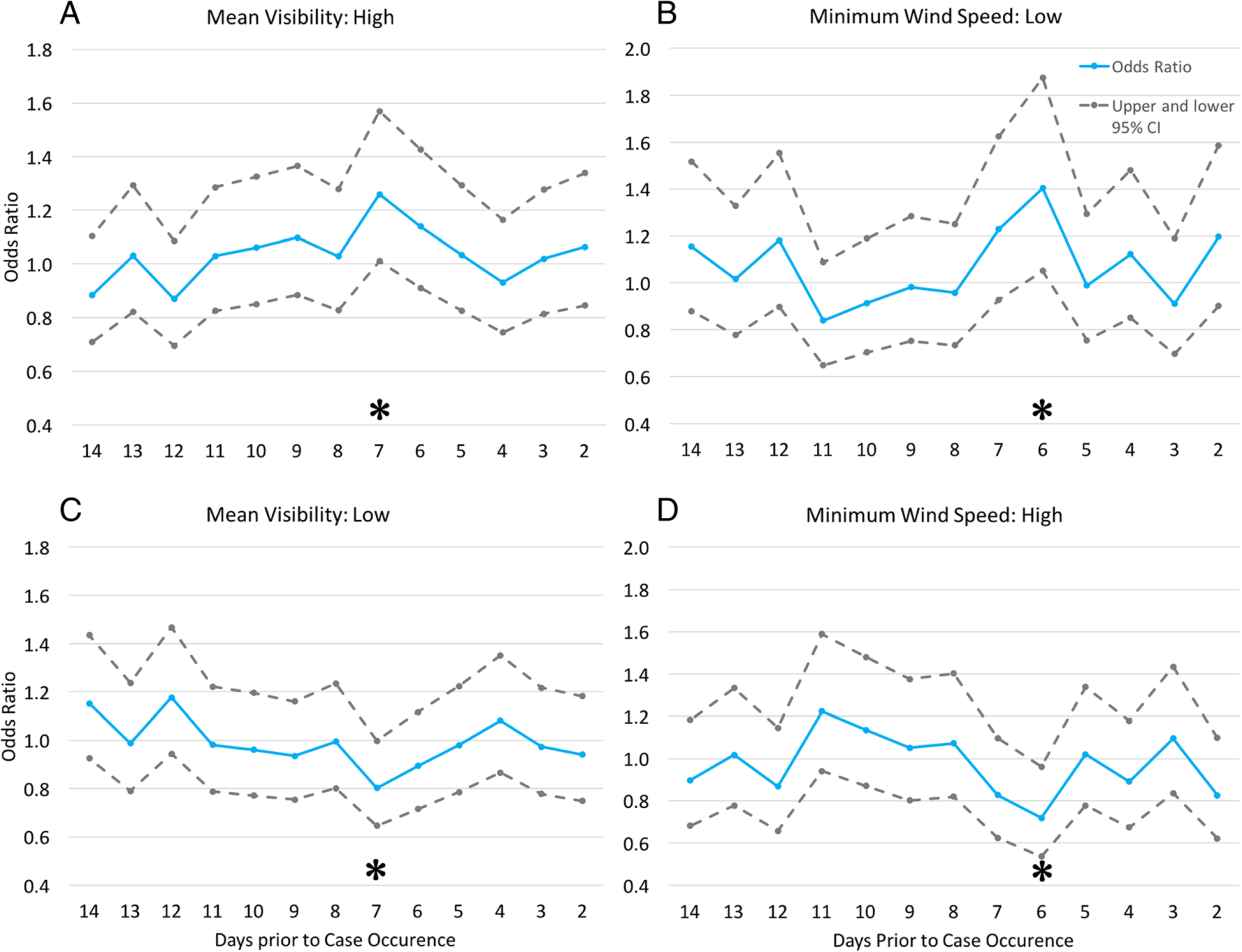
Daily visibility and wind speed variables and risk of primary MERS by province in Saudi Arabia. Odds ratio (solid line) and 95% confidence limits (dashed lines) are plotted on the Y-axis, while time lags preceding case occurrence are plotted on the x-axis. The odds of primary MERS is increased with high visibility and decreased with low visibility after 7 days (**a** & **c**), while the odds of primary MERS are increased with low wind speed and decreased when wind speed is high at 6-day lags (**b** & **d**). When maximum wind speed was high, the odds of a MERS case were increased with a 5-day lag (not shown). Asterisks indicate statistically significant odds ratios on corresponding days

High visibility was positively associated with occurrence of a MERS case 7 days later, whereas low visibility demonstrated protective effects for risk of MERS (Fig. 6). The odds of a MERS case 7 days after both minimum and mean daily visibility were high was 1.27 and 1.26 times higher than after control days (95% CI, 1.01–1.60 and 1.01–1.57). Conversely, when minimum and mean visibility were low, the odds ratios of MERS cases was 0.79 (95% CI, 0.63–0.995) and 0.80 (95% CI, 0.64–0.998).

Wind speed results were conflicting, with low minimum daily wind speed and high maximum wind speed both positively associated with cases at similar time lags (Fig. 6). The odds of a MERS case was 1.40 times higher 6 days after low minimum wind speed (95% CI, 1.05–1.87), while the odds ratio of cases for when minimum wind speed was relatively high was 0.72 (95% CI, 0.54–0.96). Conversely, the odds of a case when maximum wind speed was relatively high was 1.23 (95% CI, 1.003–1.50) (not shown in figure).

## Discussion

MERS is a global public health threat that causes severe respiratory disease with a high case fatality ratio, identified by WHO as a priority pathogen for research and development in public health emergency contexts [29]. It is primarily characterized by healthcare-associated outbreaks triggered by index cases who acquire infection from dromedary camels and possibly from unidentified asymptomatic human carriers. Improving our understanding of the epidemiology and risks of primary cases of MERS is vital for designing effective interventions that aim to reduce these index cases and prevent subsequent outbreaks in humans. The list of cases maintained by the WHO was restricted to a subset of primary cases based on explicit inclusion and exclusion criteria and was used to analyze the effect of weather on case occurrence using a case-crossover design. All four weather variables demonstrated statistically significant correlations within the incubation period for MERS in humans. The statistically significant time lags for each variable do not match up perfectly, which is to be expected and could be due to a number of reasons including natural variability in incubation periods, variable impact of weather on transmission, the interaction of unmeasured cofactors on weather variables as well the direct effect of unmeasured factors on transmission, and stochasticity in general.

Acute weather events as well as general seasonal patterns may affect disease transmission rates by altering pathogen viability and persistence in the environment as well as by influencing human behaviour and contact patterns. This study found that MERS-CoV, although a zoonotic disease, follows similar environmental transmission patterns to other non-zoonotic respiratory diseases with analogous modes of transmission such as influenza and respiratory syncytial virus. Tamerius et al. [30] have shown that global trends of influenza broadly follow either a “cold-dry” or “humid-rainy” pattern, corresponding to temperate and tropical climates. Additionally, temperate climates tend to have a single annual peak and tropical climates have semi-annual peaks. They further demonstrate that for countries with an annual influenza peak such as Saudi Arabia, temperature and humidity can be predictive of those peaks, even at latitudes close to the equator. Respiratory syncytial virus also follows similar environmental conditions, with peak timing in the Arabian Peninsula from December to February, following the distribution of cases in the temperate northern hemisphere [12]. The influence of weather is further supported by experimental evidence, which has demonstrated that lower temperatures and lower relative humidity each favour influenza transmission [11]. Furthermore, coronaviruses have been shown to exhibit strong seasonal variation in natural hosts, and the theory that these fluctuations may increase risk of zoonotic transmission at certain times of the year has been discussed [16]. The results here demonstrate that colder, drier conditions may increase the risk of zoonotic transmission of MERS from dromedaries to humans.

Sandstorms, dust storms, and air pollution in Saudi Arabia and elsewhere have been associated with increased morbidity and mortality, including from respiratory disease [31, 32]. A case-crossover study in the United States demonstrated the short-term effects of air pollution on acute lower respiratory infections [14], while another study demonstrated increased numbers of pneumonia admissions following acute dust storm events in Taiwan [33]. Dust storms can act as a pathogen carrier and also induce inflammatory reactions, potentially increasing both exposure and susceptibility to disease agents [15]. Horizontal ground visibility and wind speed were used as proxies for the occurrence of sandstorms and acute air pollution events. Visibility can be reduced to 5000 m for an average of 3.5 h during a sandstorm [34]. Summarizing the weather data used in this study, the mean daily visibility by province ranged from 82 m to over 10,000 m, although the median value was over 9000 m in all but one province. The distribution of visibility indicates that anything less than full clarity was categorized as low visibility, and that according to the measurements in [34], could indicate the presence of a sandstorm. It was hypothesized that primary MERS infections are more likely to increase following sandstorms or other severe events of air pollution that affect visibility. However, results indicate that the risk of primary MERS infection increased following high visibility days, and decreased following low visibility days. This may be due to behaviour, if people are more likely to stay inside during acute weather events, and less likely to engage in activities such as interacting with camels. It was further hypothesized that higher wind speeds would be associated with more cases of MERS. While a positive association was found between cases and high maximum wind speeds 5 days prior, there were also similar results to those of visibility. Low minimum wind speed was positively correlated with cases, and conversely, when minimum wind speed was relatively high there were statistically fewer cases of MERS. Results suggest that further investigation of wind speed as a factor for primary MERS is warranted.

There are several limitations and potential sources of bias in this study. The major cities in Saudi Arabia are severely polluted and exceed WHO guidelines, as measured by particulate matter (PM) [35, 36]. Sand and dust storms as well as other sources of air pollution such as industrial activities, fuel combustion, and traffic emissions contribute to elevated levels of PM in the country [35], all of which contribute to reduced visibility [37–39]. This study did not differentiate between sandstorms and other acute events that reduce visibility, and discerning between different forms of air pollution may provide insights about the risk of MERS-CoV transmission under different environmental conditions.

Fifty-two cases (12.3%) in the subset of primary cases had no known exposure history (no information on camel exposure, contact with a known case, nor healthcare worker status). The subset of primary cases investigated likely also include secondary cases, and is a source of selection bias. Furthermore, given that MERS is an emerging disease, case reporting and data collection standardization may have improved over the 3-year period included here.

Geographical case data were available only at the provincial level, while exposure data from the weather station closest to the largest metropolitan city in each province were used. While camel raising in the Middle East is moving from extensive to intensive production systems and concentrating around cities [40], human spillover cases would be scattered throughout the provinces to an unknown degree. Therefore, if environmental conditions differ significantly within a province, this could be a source of misclassification bias.

## Conclusions

The risk of primary human cases of MERS was associated with a decrease in temperature and humidity, and an increase in ground visibility. The temperature and humidity findings are consistent with associations between the environment and other respiratory diseases. Further study of weather and seasonal risk factors may strengthen the evidence for an environmental component of MERS-CoV transmission. A better understanding of virus viability in different environmental conditions is also a key research need. Evidence of environmental risk factors for MERS could be utilized by public or One Health practitioners for targeted interventions during higher-risk periods. The risk of MERS acquired from zoonotic transmission, or from asymptomatic carriers in the community, appears to be sensitive to weather conditions, providing key initial evidence of an environmental component for the development of primary MERS-CoV infections.

## Abbreviations

MERS-CoV: Middle East respiratory syndrome coronavirus
PM: Particulate matter
WHO: World Health Organization
NOAA: National Oceanic and Atmospheric Administration
